# Mobilization of Stem Cells Using G-CSF for Acute Ischemic Stroke: A Randomized Controlled, Pilot Study

**DOI:** 10.4061/2011/283473

**Published:** 2011-10-11

**Authors:** Kameshwar Prasad, Amit Kumar, Jitendra Kumar Sahu, M. V. P. Srivastava, Sujata Mohanty, Rohit Bhatia, Shailesh B. Gaikwad, Achal Srivastava, Vinay Goyal, Manjari Tripathi, Chandrashekar Bal, Nalini Kant Mishra

**Affiliations:** ^1^Department of Neurology, Room No. 704, Neurosciences Centre, All India Institute of Medical Sciences, Ansari Nagar, New Delhi 110029, India; ^2^Stem Cell Facility, All India Institute of Medical Sciences, New Delhi 110029, India; ^3^Department of Neuro-Radiology, All India Institute of Medical Sciences, New Delhi 110029, India; ^4^Department of Nuclear Medicine, All India Institute of Medical Sciences, New Delhi 110029, India

## Abstract

*Background*. There is emerging evidence to support the use of granulocyte colony-stimulating factor (G-CSF) therapy in patients with acute ischemic stroke. *Aims*. To explore feasibility, safety, and preliminary efficacy of G-CSF therapy in patients with acute ischemic stroke. *Patients and Method*. In randomized study, 10 patients with acute ischemic stroke were recruited in 1 : 1 ratio to receive 10 *μ*g/kg G-CSF treatment subcutaneously daily for five days with conventional care or conventional treatment alone. Efficacy outcome measures were assessed at baseline, one month, and after six months of treatment included Barthel Index (BI), National Institute of Health Stroke Scale, and modified Rankin Scale. *Results*. One patient in G-CSF therapy arm died due to raised intracranial pressure. No severe adverse effects were seen in rest of patients receiving G-CSF therapy arm or control arm. No statistically significant difference between intervention and control was observed in any of the scores though a trend of higher improvement of BI score is seen in the intervention group. *Conclusion*. Although this study did not have power to examine efficacy, it provides preliminary evidence of potential safety, feasibility, and tolerability of G-CSF therapy. Further studies need to be done on a large sample to confirm the results.

## 1. Introduction

Stroke is an important cause of mortality and morbidity worldwide [1]. Despite recent advances in antithrombotic treatment, poststroke disability has significant economic and social burden. As brain has limited capacity to regenerate, there is the need to develop therapeutic strategies to enhance neuroprotection and repair. Autologous stem-cell transplantation has been tried but has limited due to unproven efficacy and lack of available facility widespread [2].

Currently, few treatments exist for acute stroke, comprising mainly aspirin and thrombolytic drugs which have poor availability in the developing countries and very narrow time window for its intervention. A clear need exists to identify new drugs. Granulocyte colony stimulating-factor (G-CSF) is a cytokine that acts on hematopoietic stem (CD34^+^) cells and stimulates proliferation, maturation, and survival of the neutrophilic granulocyte lineage. It is widely employed to mobilize bone marrow stem cells in patients with leukaemia treated with bone marrow transplantation and chemotherapy-induced neutropenia for last two decades [3]. Since Schäbitz et al. [4] observed infarct size-reducing capabilities of G-CSF in animal stroke model, a number of preclinical investigations were initiated to explore its neuroprotective abilities. In later experimental studies of cerebral ischemia, G-CSF was found to be neuroprotective via different mechanisms, including mobilization of haemopoietic stem cells, antiapoptosis, neuronal differentiation, angiogenesis, and anti-inflammation [5, 6]. These properties are particularly significant in view of apoptosis, and inflammation has implication in the pathophysiology of cerebral ischemic injury. In virtue of the above properties, it was speculated that G-CSF not only inhibits neuron death, but also generates new neuronal tissue formation. The observation of G-CSF's effect on mobilization of stem cells from the bone marrow initiated explorations of its potential benefit in stroke with the assumption that mobilized stem cells may home into the injured brain.

Meta-analysis from the animal studies suggested that G-CSF both reduces infarct size and enhances functional recovery, and its effect is presumably dose dependent [7].

Three small clinical trials investigated the safety and feasibility and efficacy of stem cell mobilization by G-CSF in 7, 24, and 44 patients at different doses of G-CSF with acute ischemic stroke patients, respectively [8–10]. In all studies, G-CSF therapy appeared to be safe and reasonably well tolerated. Summary of G-CSF published studies in stroke patients are given in Table 1. There are several trials of G-CSF therapy in stroke ongoing across the world. Results of these trials will be helpful in knowing the efficacy of G-CSF therapy in stroke. Building on preclinical and clinical data suggesting functional and survival benefit using granulocyte colony-stimulating factor (G-CSF) in this fashion, we undertook a single centre, randomized, open-label pilot trial in patients with acute ischemic stroke. Moreover, the therapy is less invasive, relatively inexpensive (compared to rt-PA), ethically acceptable, and has long therapeutic window. The aim of the present study was to assess the safety and efficacy of G-CSFs at 10 *μ*g/kg G-CSF in patients with acute ischemic stroke and to assess the effect on circulating stem cell and blood cell counts.

## 2. Methods

### 2.1. Participants

All patients with acute ischemic stroke attending the neurology services at All India Institute of Medical Sciences, New Delhi, between January 2008 and May 2008, were screened for eligibility of this study. Patients with stroke (defined as rapidly developing clinical symptoms and/or signs of focal loss of cerebral function, with symptoms lasting more than 24 hours with no apparent cause other than that of vascular origin) were considered eligible if they fulfilled all of the following: age between 30 and 75 years, within seventh day from onset, computed tomography and/or magnetic resonance imaging scan of the brain showing no haematoma, and relevant lesions within the middle cerebral artery territory, Glasgow coma scale (GCS) score above eight (eye and motor score of more than six in patients with aphasia), Barthel index (BI) score of 55 or less, National Institute of health stroke Scale (NIHSS) score between 7 and 20, and inability to walk unaided or raise upper limb by 90 degree and clinically stable. A patient were defined as stable when they had normal respiration, was afebrile, had blood pressure less than mean arterial pressure of 125mmHg (but no hypotension defined as systolic BP <90 mmHg), and had fasting venous blood sugar level less than 200 mg% along with normal serum urea and electrolytes. 

Patients meeting the above criteria were excluded from the study if they had any one of the following: lacunar syndrome, intracranial pathologies (e.g., tumor and infection), intubation, comorbidity likely to limit survival to less than three years, for example, malignant diseases, hepatic or renal failure, prestroke disability leading to dependence on others for activities of daily living, haematological dysfunction (a history of major bleeding requiring blood transfusion or of leukopenia thrombocytopenia), inaccessibility for followup, pregnancy or unwillingness to provide written informed consent (by self or next of kin). The study was approved by the Institute Ethics Committee of AIIMS.

### 2.2. Study Design

This was a 12-month duration, randomized, open-label, parallel-group study. Eligible consenting subjects were randomly assigned in a 1 : 1 ratio to G-CSF therapy for five days along with conventional management or conventional management alone. Patients were randomly allocated to one of the two groups, by use of a computer-generated simple randomization table. The randomized allocation of groups was performed by a blinded, independent coordinator not related to patient care in the study via telephonic call. Subsequent to random allocation to groups, intervention was not blinded.

All patients were evaluated according to a protocol that included demographic data, medical history, stroke risk factors, and neurological examination. To determine stroke severity, we used the BI (scores range from 0 to 100, with lower scores indicating increasing severity) as an index of functional recovery and NIHSS score (scores range from 0 to 42, with higher scores indicating increasing severity) as an index of neurological deficit. After all of the data had been recorded, patients were randomized to receive either subcutaneous human recombinant G-CSF (filgastrim, Grafeel, India) 10 *μ*g/kg subcutaneously administered daily for five days along with conventional treatment or conventional treatment alone. Intervention was given within two hours of randomization. We assessed safety of subcutaneous G-CSF infusion by recording the development of immediate or delayed reactions. Immediate reactions included allergic reactions (tachycardia, fever, skin eruption, and leukocytosis). Leukocyte counts were measured on day one, three, five, and seven from blood samples. One week after the initiation of therapy, patients were discharged unless clinically warranted.

After discharge, all of the patients were followed up at one month; modified Rankin scale (scores range from zero to six, with higher scores indicating increasing severity) as an index of functional recovery, along with BI and NIHSS, was recorded. Adverse events elicited included bone pain, headache, liver dysfunction, myocardial infarction, recurrence of stroke, and peripheral arterial thromboses. Subsequently, all of the patients were followed up at six and twelve months in the outpatient department, and neurological functions were assessed using all three scales. The 12-month scores from the BI, NIHSS, and mRS were used to assess treatment efficacy. Improvement was defined as the percentage change in mean group scores between baseline and 12 months. To evaluate tumor formation as a delayed complication, we performed a regular physical examination including visual inspection of skin and oral mucosa, a follow-up magnetic resonance imaging brain at one month and six-months, and whole body FDG-positron emission tomography at the end of 12 months.

### 2.3. Statistical Analysis

Analysis was done after completion of six-month followup. The primary analyses of efficacy and safety were performed on the intention to treat analysis. This included all patients who were randomized to receive treatment. Baseline characteristics and differences between G-CSF and control groups with different outcome measures were analyzed using Mann-Whitney U-tests. Data was analyzed using the SPSS statistical package, version 17.0 (SPSS, Chicago, IL, USA).

## 3. Results

**Figure 1:**
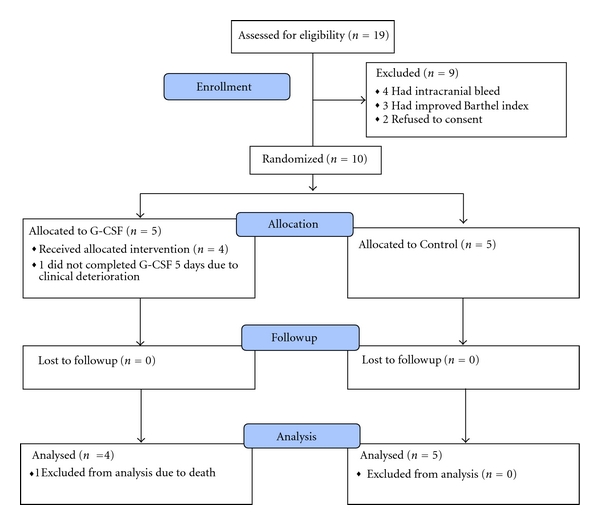
Enrolment, randomization, and analysis of patients.

Between January 2008 and May 2008, a total of 19 patients with acute ischemic stroke were screened for eligibility (Figure 1). Of those 19 patients, nine were excluded, as four had intracranial bleed, three had improved Barthel index, and two refusals to consent by caregivers. All ten consecutive patients who were found eligible were randomly assigned to G-CSF or control. At baseline, five patients were in G-CSF group and five patients were in control group. In G-CSF group, all patients except one completed 5-day course of G-CSF therapy. One patient had clinical deterioration on day three of G-CSF therapy, so intervention was withheld, and patient died on eighth day after randomization. None of the patients showed deterioration in NIHSS, BI, or mRS during the followup. No patient developed liver or renal dysfunction. PET scan at one year did not show any evidence of tumor formation.

As per protocol numbers of patients completed trial were four in G-CSF arm and five in control arm. There were no losses to followup over the study period. Baseline characterises of individual patients is summarized in Table 2. There was no significant difference in baseline characteristics between the two groups (Table 3). 

**Figure 2:**
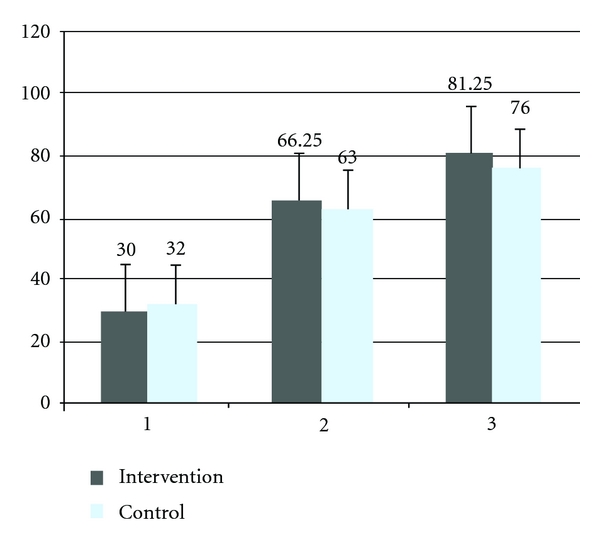
Mean Barthel index scale score at baseline, one month and six months of the intervention and control group. 1; baseline, 2; one month, 3; six months, BI scores range from 0 to 100, with lower scores indicating increasing severity.

**Figure 3:**
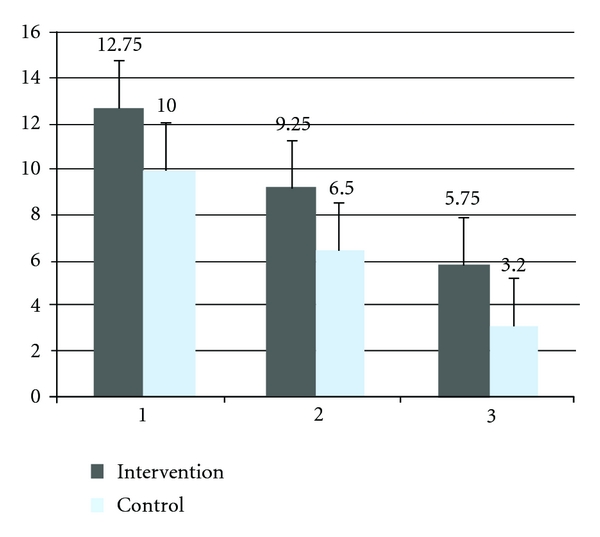
Mean National Institute of Health scale score, at baseline, one month and six months of the intervention and control group. 1; baseline, 2; one month, 3; six months, NIHSS scores range from 0 to 42, with higher scores indicating increasing severity.

**Figure 4:**
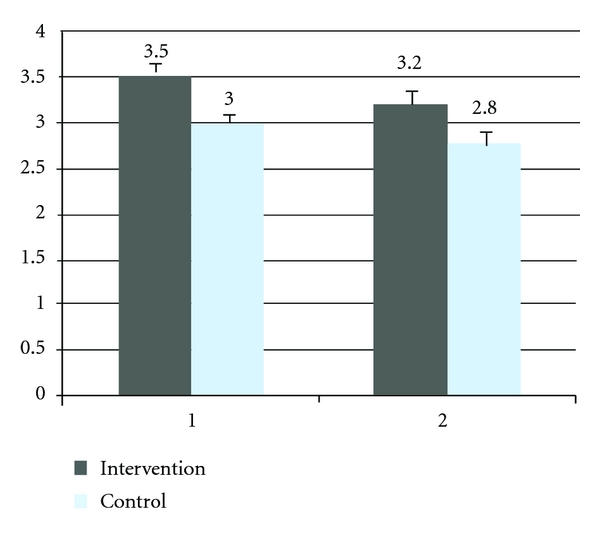
Mean modified Rankin scale score at one month and six months of the intervention and control group. 1; one month, 2; six months, mRs score range from 0 to 6, with higher scores indicating increasing severity.

Improvement in BI, NIHSS, and mRS did not differ significantly between the G-CSF and control groups, Figures 2, 3, and 4. Although a trend of higher improvement of BI score is seen in the intervention group, the difference did not achieve statistical significance. 

**Figure 5:**
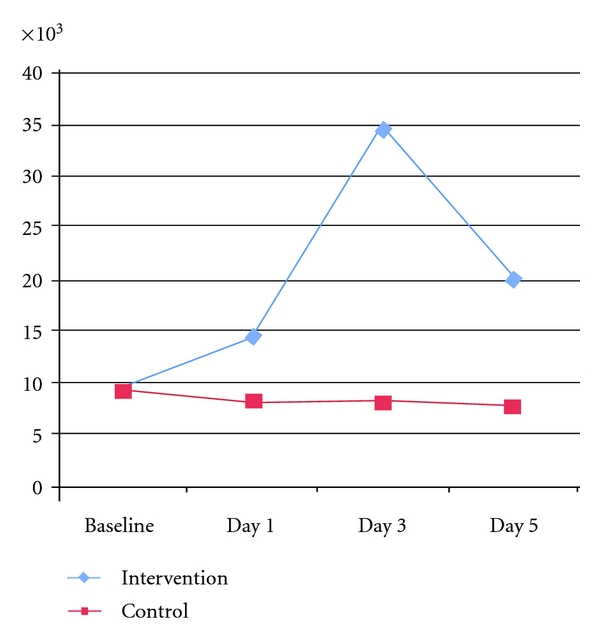
Mean Leukocyte count of intervention and control groups at Baseline, Day 1, Day 3, and Day 5.

Rise in maximum leukocyte count at hospital stay from baseline and clinical outcome score in individual patients wise assessed at different time interval is given in Table 4. There was statistically significant rise in the mean leukocyte count and alkaline phosphatase levels in the intervention arm as compared with baseline and to control arm (*P* < 0.005 and 0.01, resp.). Rise in mean leukocyte is represented in Figure 5. Rise in peripheral blood CD-34 count did not differ significantly between the G-CSF and control groups.

G-CSF therapy was reasonably well tolerated. Of the five patients receiving G-CSF, one reported mild bone pain that lasted one day and subsided spontaneously. One patient in G-CSF arm had deep venous thrombosis in left lower limb that required hospitalisation and subsided with treatment. There were no aggravations of stroke symptoms during the course of therapy and hospital stay. There was no aggravation of limb weakness, speech impairment, or sensory impairment during the 12-month followup in either study group. No severe adverse effects were seen in any of patients receiving G-CSF therapy arm or control arm.

## 4. Discussion

This is the first preliminary randomized controlled study to explore safety and preliminary efficacy of G-CSF in patients with stroke from India. In accordance with previous studies, we found G-CSF therapy safe and well tolerated in five patients with acute stroke [8–10]. 

We failed to find effectiveness of G-CSF in improving neurologic outcome in patients with acute ischemic stroke. With only five patients in each arm, the pilot study was not designed to have the power to detect outcome differences between the groups. 

Two points deserve some comments.

1. *Optimal dose of G-CSF*: several studies have been completed at different doses of G-CSF. (Table 1.) Our study suggest that G-CSF at dose of administered 10 *μ*g/kg daily for five days appeared to be safe and reasonably well tolerated, and there was higher trend of improvement in Barthel index score in intervention group compared to control. Previous studies [10, 11] have used 10 *μ*g/kg daily for five days protocol. To allow comparison of our results with the published studies and in future to facilitate meta-analyses, we have followed the same protocol. Several studies are ongoing with different doses of G-CSF in stroke and results of all studies will be helpful to determine the dose-response gradient of G-CSF. CD-34^+^ cells in peripheral blood increases significantly after G-CSF injection of 10 *μ*g/kg for consecutive five days [10]. There was dose-dependent beneficial effect observed in treatment with patient with DWI lesion >14–17 cm^3^ [8]. Our study provide basis for the second trial with the dose of 10 *μ*g/kg daily for five days. In view of similar findings from other G-CSF trials [8–10] in stroke patients, G-CSF therapy appeared safe and reasonably well-tolerated. Our data also suggest that rise in the leukocyte and alkaline phosphatase at this dose is not challenging in acute stage of stroke. There is clear need for identification of optimal dose of the G-CSF at which its effects is highest in improving functional outcome in stroke. More studies are needed to determine the optimal dose of G-CSF.
2. *Timing of G-CSF injection*: a therapeutics time window for intervention is a major promise of G-CSF therapy. Time of intervention used in clinical studies is summarised in Table 1. At present-time treatment for stroke, particularly thrombolytic therapy with tissue plasminogen activator is challenging because of its short time window of efficacy therefore, there is a clear need for novel and effective treatment options, with a longer time window. Shyu et al. [9] tested within seven days of onset of stroke and found there were consistent trend towards improvement in neurological functional recovery in G-CSF group. CD34^+^ stem cells also effectively mobilized and appeared to be safe and well tolerated after G-CSF injection when treatment is delayed for month in ischemic stroke patients [10]. Wider therapeutic window would be a significant achievement for stroke, since patients often does not reach hospital—nor is the disease often diagnosed until later than 3 hours after onset. Schäbitz et al. [8] 2010 (AXIS trial) tested within 12 hours of onset of stroke and found it is well tolerated and more effective with higher doses in patients with larger lesion volume. Timing of GCSF administration is likely to influence its neuroprotective effects. Mechanism of action of G-CSF may primarily on neurons; it is likely that the earlier treatment may have more potent neuroprotective effect. Our study suggests that treatment with G-CSF within seven days of onset of stroke appeared to be safe and reasonably well tolerated.

Based on the findings of the pilot study, Drug-Controller-General of India has approved our multicentric, phase I/II safety and efficacy, randomized controlled trial of G-CSF with 200 sample size of patients. Our planned clinical trial is to establish safety and explore efficacy of G-CSF therapy in acute stroke patients. The protocol is under consideration for funding in the Department of Biotechnology, Government of India.

**Limitation of Study**

The study is with small number of sample size; however, other studies are ongoing and published in international journals with small number of sample sizes.

In conclusion, G-CSF administered 10 *μ*g/kg daily for five days appeared to be safe and well tolerated in five patients aged 35–75 years with acute ischemic stroke in accordance with other published G-CSF trials in stroke patients. However, in view of limited sample size, the results of the current study must be interpreted with caution. Further, large, adequately powered, multicenter randomized placebo-controlled, blinded trials are needed to test the efficacy of G-CSF in patients with acute ischemic stroke.

## Figures and Tables

**Table 1:** Summary of published studies of G-CSF therapy in stroke patients.

Author (year)	Trial design/phase	G-CSF regimen	Time after stroke	Patients (intervention/control)	Comments
Floel et al. (2011)	Randomized controlled trial	10 *μ*g/kg s/c for 10 days	4 months after	21 Intervention 20 Placebo	Feasibility and safe and reasonable tolerable in chronic stroke patients
Schäbitz et al. (2010)	Randomized, placebo-controlled	30 *μ*g/kg, 90 *μ*g/kg, 135 *μ*g/kg, 180 *μ*g/kg	Within 12 hours	14/ Placebo 8/30 *μ*g/kg 7/90 *μ*g/kg 8/135 *μ*g/kg 7/180 *μ*g/kg	Well tolerated even in higher doses and Treatment effect in patients with higher volume of lesion size (*˃*14–17 cm^3^) at baseline
Shyu et al. (2006)	Single blind controlled/pilot	15 *μ*g/kg/day s/c for 5 days	Within 7 days	7 Intervention 15 *μ*g/kg/day s/c for 5 days 3 Control	No thrombotic complications, and improved outcome in G-CSF group NIHSS 59% in G-CSF group, 36% in controls group BI 120% in G-CSF group, and 60% in controls group
Sprigg et al. (2006)	Double-blind placebo-controlled/pilot	Dose escalation 1–10 *μ*g/kg s/c for 1 or 5 days	7–30 days	12/Placebo 4/1 *μ*g/kg (single dose) 4/3 *μ*g/kg (single dose) 4/10 *μ*g/kg (single dose) 4/1 *μ*g/kg (five dose) 4/3 *μ*g/kg (five dose) 4/10 *μ*g/kg (five dose)	No difference in SAEs although non significant increase in infection rates in active group Significant increase in CD-34^+^ with 10 *μ*g/kg (five dose) at day five
Zhang (2006)	Double-blind placebo-controlled/pilot	2 *μ*g/kg/day s/c for 5 days	Within 7 days	15 Intervention 30 Control	No difference in adverse events reported and significant reduction in NIHSS

**Table 2:** Patient's characteristics.

Case no.	1	2	3	4	5	6	7	8	9	10
Allocation	Control	G-CSF	Control	G-CSF	G-CSF	G-CSF	Control	Control	Control	G-CSF
Days b/w onset of stroke and randomization	3	4	7	4	4	3	6	2	1	5
Age/sex	56	45	35	45	40	55	55	65	30	38
Territory and side	Rt MCA	Rt MCA	Lt MCA	Lt MCA	Rt MCA	Both MCA & ACA	Rt MCA	Lt MCA	Lt MCA	Rt MCA
Previous stroke	−	−	−	−	−	−	−	**+**	−	−
GCS baseline	15.00	15.00	15.00	14.00	15.00	14.00	15.00	11.00	15.00	15.00
Hypertension	+	−	−	−	−	+	−	−	−	−
Diabetes	−	−	−	−	−	−	−	−	−	−
Dyslipidemia	−	+	−	−	−	+	−	−	−	−
Smoker	−	−	−	−	−	+	−	+	−	−

**Table 3:** Summary of patient characteristics and efficacy outcomes (difference between six-month and baseline scores).

	Intervention *n* = 5	Control *n* = 5	*P*-value
Age in years*	44 ± 6.5 (38 to 55)	48 ± 14.9 (30 to 65)	0.63
GCS score*	14.7 ± 5 (14 to 15)	14.2 ± 1.7 (11 to 15)	0.65
Days b/w onset of stroke and randomization*	4 ± 2.44 (1 to 7)	4 ± 0.7 (3 to 5)	0.87
NIHSS score^∗†^	14 ± 3.9 (8 to 19)	11.0 ± 3.39 ( 7 to 15)	0.23
Mean difference of NIHSS from six months to baseline	−7.0	−6.75	0.90
BI score^∗‡^	25 ± 17.67 (5 to 45)	32 ± 21.09 (15 to 55)	0.58
Mean difference of BI from Six months to baseline	51.25	44	0.59
Mean difference of mRS from six months to one month	−0.5	−0.4	0.79

*Figures represent mean ± SD (range) or numbers.

^†^NIHSS scores range from 0 to 42, with higher scores indicating increasing severity.

^‡^BI scores range from 0 to 100, with lower scores indicating increasing severity.

^§^mRs score range from 0 to 6, with higher scores indicating increasing severity.

**Table 4:** Leukocyte counts at baseline and maximum count during hospital stay and clinical score during baseline one month, six months, and 12 months.

	Total leukocyte count		Stroke scale scores at baseline, one month/six months and 12 months		
Patient no.	Baseline	Maximum during hospital stay	NIHSS	BI	mRS
G-CSF group					
1	13400	40800	14/10/9	10/35/65	4/4
2	12400	—	19/−/−	5/−/−	−/−
3	7500	35400	14/9/6	45/80/85	3/3
4	7600	39200	15/7/3	25/90/95	3/2
5	8400	33400	8/9/5	40/60/80	4/3

Control group					
1	7000	7000	13/10/5	15/35/55	4/4
2	13500	13500	12/10/4	55/60/75	4/3
3	8500	10400	15/12/−	20/30/55	4/4
4	7300	7300	7/2/2	55/100/100	2/1
5	10100	10100	8/4/2	15/90/95	2/2

NIHSS: National Institutes of Health stroke scale, BI: Barthel index, mRS: modified ranking scale

NIHSS score range from 0 to 42 (lower score represents better outcome and higher score represents worse outcome)

BI score range from 0 to 100 (Higher score represents the better outcome and lower score represents the worse outcome)

mRS score range from 0 to 6 (lower score represents better outcome and higher score represents worse outcome).
